# Supplementary material for: Frequency doubling in the cyanobacterial circadian clock
Source: Mol Syst Biol. 2016 Dec 22;12(12):896. doi: 10.15252/msb.20167087 (PMC5199125; doi:10.15252/msb.20167087)
Supplement: Supplementary file 1 — Appendix [file MSB-12-896-s001.pdf]

Frequency doubling in the cyanobacterial circadian clock

Bruno M.C. Martins[1+], Arijit K. Das[1+], Liliana Antunes[1,2], James C.W. Locke[1,3,4*]

[1]Sainsbury Laboratory, University of Cambridge, Bateman Street, Cambridge CB2 1LR, UK

[2]Wellcome Trust Sanger Institute, Hinxton, Cambridge CB10 1SA, UK

[3]Department of Biochemistry, University of Cambridge, Cambridge CB2 1QW, UK

[4]Microsoft Research, 21 Station Road, Cambridge CB1 2FB, UK

Appendix

Table of contents

I. Appendix figures 2

II. Mathematical model 9

III. Generation of double peaks from an activator-repressor gate 12

I. Appendix figures

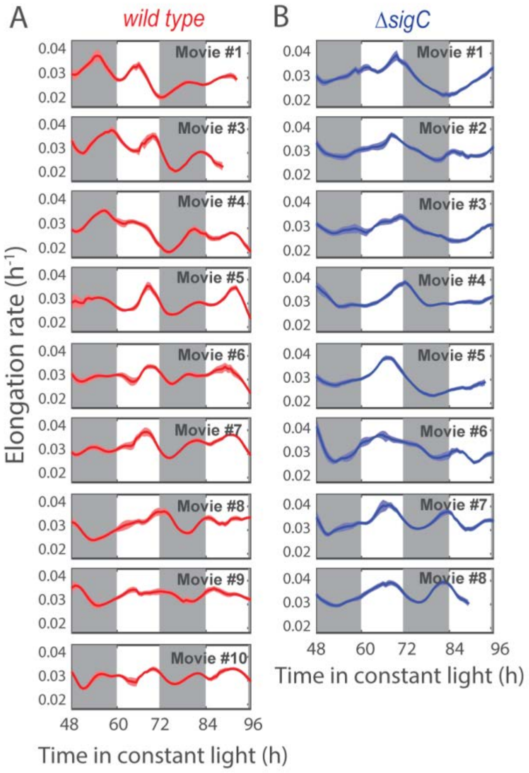

Figure S1: **Two peak circadian oscillations in growth can be observed in wild type cells**.

A. Mean of individual cell elongation rates from nine movies of P_{psbAI}-YFP reporter in WT background between 48 and 96 hours. Movie #2 not shown as it ended after just 80 hours. Daily double peaks of growth rate can be observed. The pink shade represents the standard error of the mean.

B. Mean of individual cell elongation rates for eight movies of *sigC* deletion strains. Single peak circadian oscillations in growth can be observed in the majority of cases. The blue shade represents the standard error of the mean.

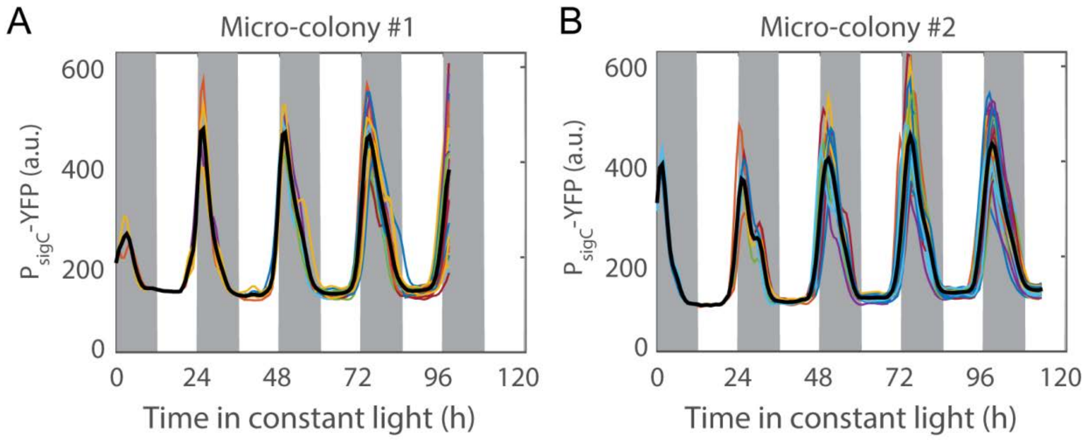

Figure S2: **Single cell lineages from two movies of *sigC* expression show single peak oscillations**.

A-B. Time traces of P_{sigC}-YFP reporter strains grown under low light (ca. 15 $\mu E\ m^{-2}\ s^{-1}$ cool white light). Individual lineages show high amplitude narrow peaks and an absence of frequency doubling. The black line represeants the mean across all single cell traces.

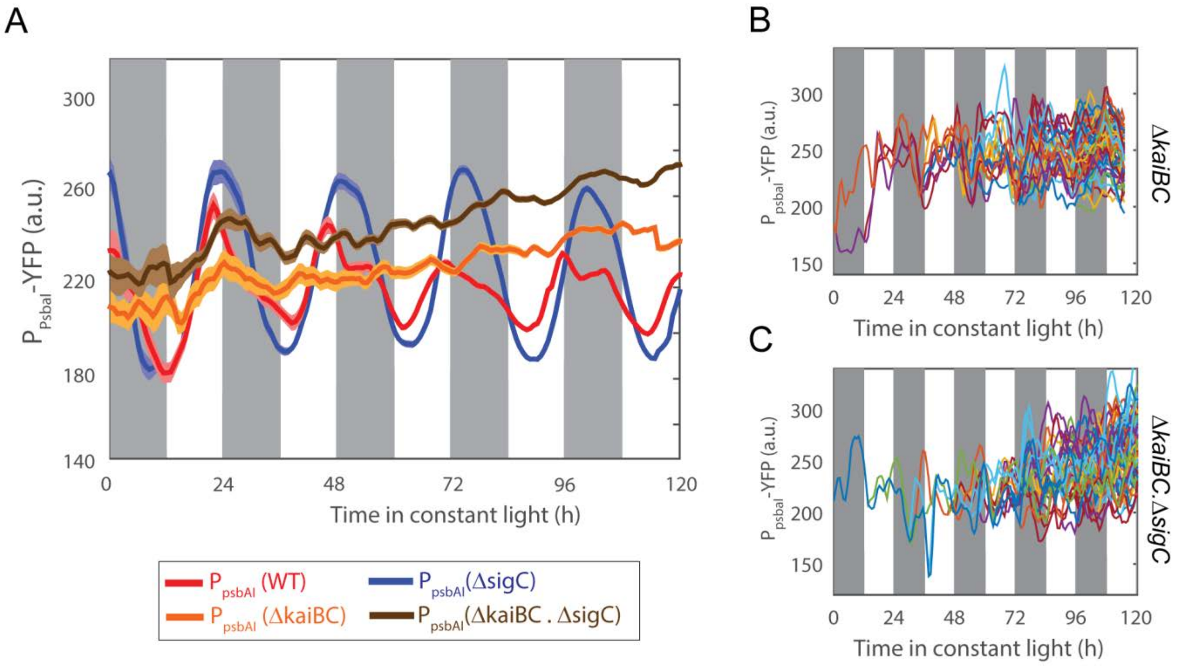

Figure S3: **Expression of P_{psbAI}-YFP in mutant backgrounds reveals that oscillatory dynamics are due to *sigC* and the circadian clock.**

A. Mean traces of P_{psbAI}-YFP in WT (red), *sigC* deletion (blue), *kaiBC* deletion (orange), and *kaiBC-sigC* double deletion backgrounds (brown). For the wild type strain, 1319 cells from 10 movies (with up to 419 cells per time point) were collected. For the *sigC* deletion strain, 1088 cells from 8 movies (with up to 419 cells per time point) were collected. For the *kaiBC* deletion strain, 1332 cells from 8 movies (with up to 501 cells per time point) were collected. For the *kaiBC-sigC* double deletion strain, 2249 cells from 12 movies (with up to 641 cells per time point) were collected. The colour shades represent standard errors of the mean. Circadian oscillations are abolished in *kaiBC* deletion and *kaiBC-sigC* double deletion backgrounds.

B-C. Fluctuations are observed in single cell lineages in a *KaiBC* deletion background (B) and in a *kaiBC-sigC* (C) double deletion background.

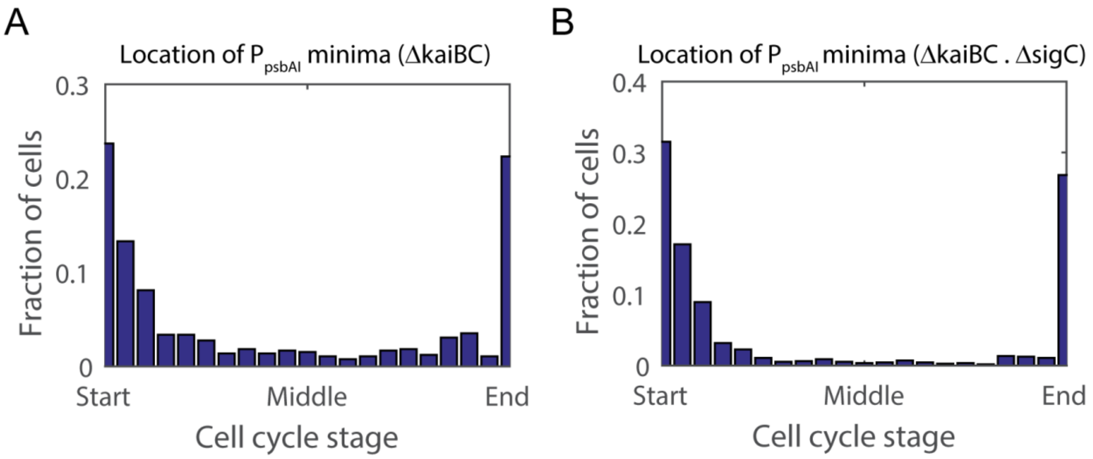

Figure S4: **Location of YFP minima along the cell cycle for P_{psbAI}-YFP**.

A-B. The location of expression minima reveals that fluctuations observed in *kaiBC* deletion (A) and *kaiBC-sigC* double deletion (B) strains are due to dips in expression occurring at around the time of division.

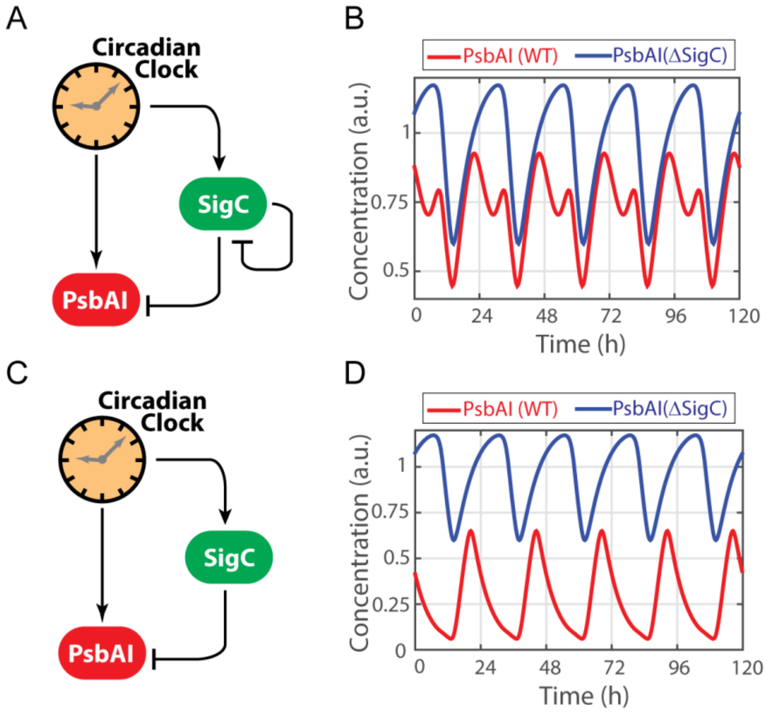

Figure S5: **The negative feedback of SigC on itself enhances double peaks.**

A-D. The negative auto-regulatory function of SigC (A, C) provides an additional level of control to allow a doubling of the frequency of peaks of expression in numerical simulations (B, D). Other than removing the feedback, the same parameter set was used in the simulations in (B) and (D).

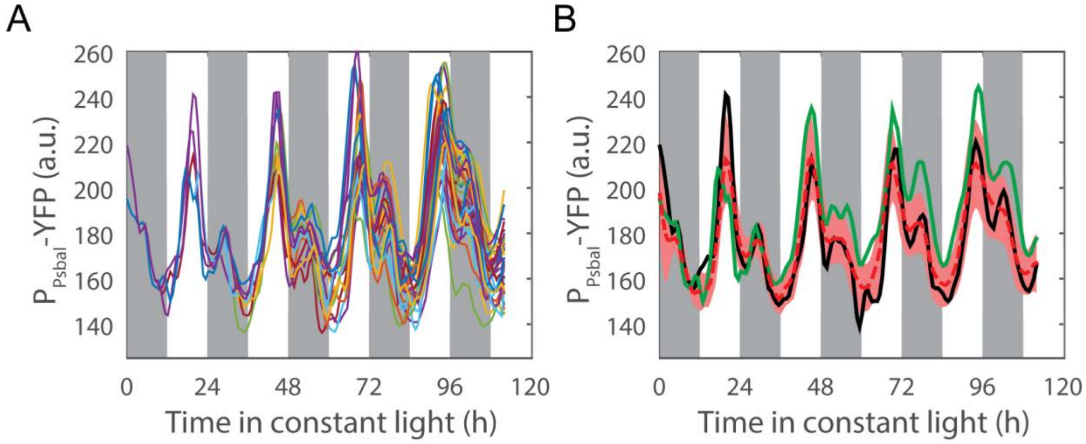

Figure S6: **Single cell lineages from one movie of *psbAI* expression under very low light conditions show two peak oscillations.**

A. Time traces of P_{psbAI}-YFP reporter grown under very low light (ca. 10 μE m^{-2} s^{-1} cool white light). Individual lineages show the existence of a secondary peak following the main (dusk timed) peak of expression.

B. Two representative traces (green and black) display double peaks, but due to desynchronisation, a double peak is less apparent in the mean trace (red dotted line). Pink shading represents one standard deviation from the mean. 215 cells from 3 movies (with up to 104 cells per time point) were collected.

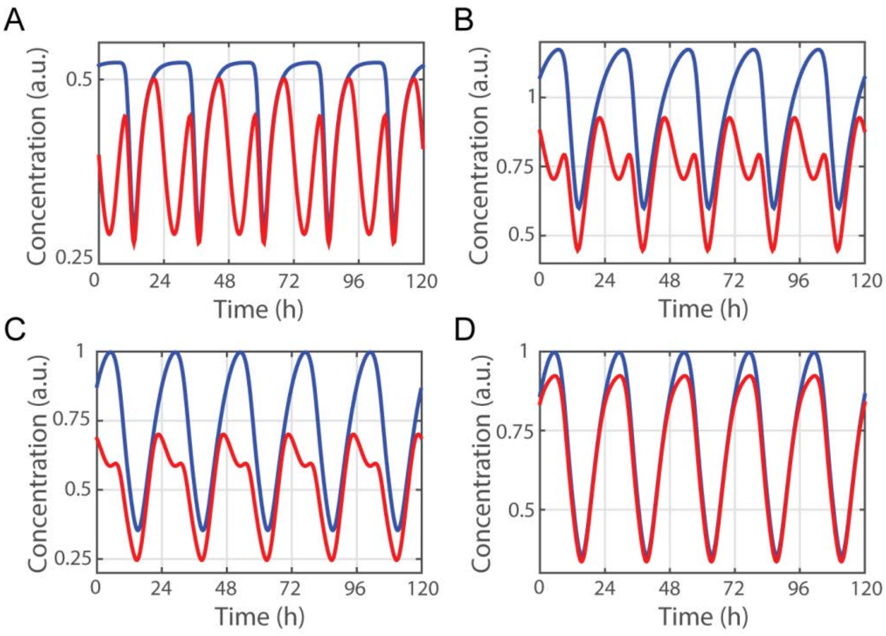

Figure S7: **The oscillatory incoherent feed forward loop model can generate a variety of oscillatory dynamics**.

A-D. Numerical simulations show the strength of the double peak can be tuned from two near equal peaks (A) to asymmetric double peaks (B), shoulders (C), and single peaks (D). In all panels, the red line represents the wild type and the blue line represents the SigC deletion mutant.

II. Mathematical model

The model used in this study is a minimal phenomenological model translated into ordinary differential equations (ODEs). Our purpose is to gain an understanding of the principles that underlie the generation of a doubling in the frequency of expression peaks in oscillatory circuits, such as we observed *in vivo*. We therefore chose not to model the circadian clock explicitly, and to simplify the regulatory terms. The species in the model are the circadian clock, PsbAI (or RpoD6), and SigC (which we split into active and inactive forms).

We describe the time evolution of the clock, Θ, by a sinusoidal signal of the form:

$$\Theta(t) = b + \frac{1}{2}(A - b)\left(1 + cos\left(\frac{2\,\pi\,t}{T_C}\right)\right), \tag{S1}$$

where T_C = 24 h, b is the basal level of the clock signal and A is its maximum.

This clock signal then regulates expression of its targets. For simplicity, we model only transcription and assume the signal Θ directly regulates PsbAI (or RpoD6) and SigC. We represent this regulation using Hill equation kinetics. In our scheme (Figure 4A in the main text), we postulate SigC negatively regulates PsbAI and itself. Whether the repressing activity of SigC is direct or indirect remains a question to be addressed in the future. In our model, and again in the interest of simplicity, we also represent the activity of SigC by Hill equation kinetics. The total production rate therefore depends on two variables – the clock and SigC – and incorporates 4 possible states of the "promoter": no regulator is "bound"; only the clock output is "bound"; only SigC is "bound"; and both regulators are "bound". The total production rate f_X then becomes:

$$f_X = V_X \frac{\left(\frac{\Theta}{K_{\Theta X}}\right)^{h_{\Theta X}}}{1+\left(\frac{\Theta}{K_{\Theta X}}\right)^{h_{\Theta X}}+\left(\frac{[SigC_a]}{K_{SX}}\right)^{h_{SX}}+\left(\frac{\Theta}{K_{\Theta X}}\right)^{h_{\Theta X}}\left(\frac{[SigC_a]}{K_{SX}}\right)^{h_{SX}}}, \tag{S2}$$

where V_X is the maximal production rate; $K_{\Theta X}$ is the activation coefficient of species X by the clock; $h_{\Theta X}$ is the degree of cooperativity of that activation; K_{SX} is the repression coefficient of species X by

SigC (by its active form $SigC_a$); h_{SX} is the degree of cooperativity of that repression; and $[SigC_a]$ is the concentration of the active form of SigC (the only form that can affect expression of downstream targets). We normalise our model by dividing all species, maximal rates and K coefficients by the maximal output of the clock (A). This operation means the system is effectively modelled in units of clock output.

The system of ODEs is:

$$\frac{d[PsbA1]}{dt} = V_P \frac{\left(\frac{\Theta}{K_{\Theta P}}\right)^{h_{\Theta P}}}{1+\left(\frac{\Theta}{K_{\Theta P}}\right)^{h_{\Theta P}}+\left(\frac{[SigC_a]}{K_{SP}}\right)^{h_{SP}}+\left(\frac{\Theta}{K_{\Theta P}}\right)^{h_{\Theta P}}\left(\frac{[SigC_a]}{K_{SP}}\right)^{h_{SP}}} - \log(2)\left(\frac{1}{T_d}+\frac{1}{T_Y}\right)[PsbA1], \qquad \text{(S3)}$$

$$\frac{d[SigC]}{dt} = V_S \frac{\left(\frac{\Theta}{K_{\Theta S}}\right)^{h_{\Theta S}}}{1+\left(\frac{\Theta}{K_{\Theta S}}\right)^{h_{\Theta S}}+\left(\frac{[SigC_a]}{K_{SS}}\right)^{h_{SS}}+\left(\frac{\Theta}{K_{\Theta S}}\right)^{h_{\Theta S}}\left(\frac{[SigC_a]}{K_{SS}}\right)^{h_{SS}}} - \log(2)\left(\frac{1}{T_d}+\frac{1}{T_S}\right)[SigC] - k_f[SigC] +$$

$$+ k_b[SigC_a], \qquad \text{(S4)}$$

$$\frac{d[SigC_a]}{dt} = -\log(2)\left(\frac{1}{T_d}+\frac{1}{T_S}\right)[SigC_a] + k_f[SigC] - k_b[SigC_a]. \qquad \text{(S5)}$$

In equations (S3) and (S4), the parameters in the production rate terms are as described in Equation (S2) (with the subscripts P and S representing $PsbA1$ and $SigC$ respectively). In the dilution terms, T_d is the average cell cycle duration (and so it represents dilution by cell growth), T_Y is the reported half-life of the fluorescent reporter used in the experiments (YFP_LVA) (Chabot et al, 2007), and T_S is half-life of of SigC (we assume both forms of SigC are unstable and have the same half-life). k_f and k_b are the forward and back rates of activation of SigC. This reaction may, for example, represent binding and unbinding of SigC to the RNA polymerase. We choose to include this reaction in our system in order to be able to model the possible effects of environmental perturbations in the activity of SigC.

The SigC deletion mutant is simulated by eliminating the two last terms in the denominator of the production rate, i.e.,

$$\frac{d[PsbA1^{mut}]}{dt} = V_P \frac{\left(\frac{\Theta}{K_{\Theta P}}\right)^{h_{\Theta P}}}{1+\left(\frac{\Theta}{K_{\Theta P}}\right)^{h_{\Theta P}}} - \log(2)\left(\frac{1}{T_d}+\frac{1}{T_Y}\right)[PsbA1^{mut}]. \quad \text{(S6)}$$

$RpoD6$ is modelled by an equation equivalent to Equation (S3), updating the respective parameters.

To generate the numerical simulations presented in the main text and supplementary figures, the initial conditions of all species are set to 0. The system is simulated for 600 h, but only the final 120 h are shown with t=0 scaled accordingly. In Figure 4 we used the following set of parameters:

Table S1: Parameters used for the simulations in Figure 4.

**Parameter**	**Description**	**Value (in units of clock output)**
b	basal level of the clock	0
A	maximal level of the clock	1
V_S	maximal production rate of SigC	3.7 h^{-1}
$K_{\theta S}$	activation coefficient of SigC by the clock	0.9
K_{SS}	repression coefficient of SigC by itself (by its active form $SigC_a$)	1.4
$h_{\theta S}$	degree of cooperativity on the activation of SigC by the clock	5
h_{SS}	degree of cooperativity on the repression of SigC by itself (by its active form $SigC_a$)	2
V_P	maximal production rate of PsbAI (or RpoD6)	0.2 h^{-1}
$K_{\theta P}$	activation coefficient of PsbAI (or RpoD6) by the clock	0.1
K_{SP}	repression coefficient of PsbAI (or RpoD6) by $SigC_a$	2.5
$h_{\theta P}$	degree of cooperativity on the activation of PsbAI (or RpoD6) by the clock	2
h_{SP}	degree of cooperativity on the repression of PsbAI (or RpoD6) by $SigC_a$	5
T_d	mean cell cycle time	19.5 h
T_Y	reporter half-life	5.6 h
T_S	SigC half-life	6 h
k_f	activaton rate of SigC	10 h^{-1}
k_b	deactivaton rate of SigC	0.1 h^{-1}

To generate the simulations for RpoD6 shown in Figure 6D, all parameters were kept the same except the activation coefficient $K_{\Theta P}$, which was set to 0.3. This parameter describes a characteristic

of the PsbAI and RpoD6 "promoters" (e.g., its sequence), and so it is reasonable to assume this is one parameter biological systems can tune to generate different frequency modulation.

To generate the numerical simulations for the higher light perturbation (Figure EV3A, dashed line in Figure EV3B), we modified the cell cycle duration, T_d, to 9.3 h (the value we measured experimentally), and the back rate k_b to 10 h^{-1}. The assumption underlying this modification is that SigC is less active (or more unstable) at higher light, which is a possible interpretation of our observations (Figure 5, Figure EV2, Figure EV3). We note that other modifications could be responsible for the near disappearance of the double peak in the experiments (Figure 5A). For example, the amplitude of the clock may change under different light conditions (through parameters b and A), which may also move the output of the model from a double peak to a single peak result. However, they do not result in the upregulation of sigC expression observed experimentally (Figure EV2). In Figure EV3B, the sum of the two forms of SigC ($SigC$ and its active form $SigC_a$) is shown. The low light simulation (solid red line, Figure EV3B) uses the parameters from Table S1.

Finally, for the simulations shown in Figure S7 (and in inset of Figure 6D), we made the following changes to the parameters in Table S1: in panel (A) $b = 0.08$, $h_{SS} = 1$, $h_{\Theta P} = 5$, $h_{SP} = 4$, $T_{\mathrm{Y}} = 2$ h, $T_{\mathrm{S}} = 3$ h; in panel (B) no changes were made; in panel (C) $K_{\Theta P} = 0.3$; in panel (D) $K_{\Theta P} = 0.3$, $K_{SP} = 4$.

III. Generation of double peaks from an activator-repressor gate

1. Double peaks in production rate. Our network motif contains an output that is regulated by an oscillatory positive regulator and an oscillatory negative regulator, as described before. It can be

shown that a general model of the kind described by Equation (S2) is intrinsically a two peak model. Previous studies have already demonstrated that two oscillatory inputs coupled non-linearly (e.g., an AND gate where the output is the product of the two inputs) can generate sub-circadian harmonics (Westermark & Herzel, 2013). In our model we have a production rate given by:

$$f = V \frac{u_1}{1+u_1+u_2+u_1\, u_2}, \qquad \text{(S7)}$$

where u_1 and u_2 are contributions from inputs 1 (a positive regulator) and 2 (a negative regulator) respectively, where

$$u_j = \frac{1}{K_{M,j}} \gamma_j \left(1 + a_j\, cos\big(\omega\, (t + \phi_j)\big)\right). \qquad \text{(S8)}$$

Here, $\gamma_j = \left(A_j + b_j\right)/2$, $a_j = \left(A_j - b_j\right)/\left(A + b_j\right)$, $K_{M,j}$ is a Michaelis-Menten constant, ω_j is the angular frequency, and ϕ_j is a phase displacement. The terms γ_j and a_j are only added to ensure the input oscillates between a basal level $b_j > 0$ and a saturation level A_j. We drop the Hill coefficients because if we can show Michaelis-Menten kinetics can generate double peaks, then the added non-linearity of the Hill equation should only reinforce that output. Two peaks per circadian cycle are possible when the two oscillatory inputs are out of phase (Westermark & Herzel, 2013). We will consider the more restrictive case where they have the same phase ($\phi_j = 0$).

In our model, double peaks of expression require double peaks of production rate. We can find the double peaks by solving $\frac{df}{dt} = 0$. In other words, we need to find the roots of the numerator of $\frac{df}{dt}$:

$$sin(\omega\, t)\, \left[a_2\, g_2(1 + g_1) - a_1\, (1 + g_2) + a_1 a_2\, g_1\, g_2\, cos(\omega\, t)\big(2 + a_1\, cos(\omega\, t)\big)\right] = 0, \qquad \text{(S9)}$$

where $g_j = \gamma_j / K_{M,j}$. Since one of the terms is simply $sin(\omega\, t)$, then one pair of roots is $t = \left\{\frac{2\pi\, n}{\omega}, \frac{\pi + 2\pi\, n}{\omega}\right\}, n \in \mathbb{Z}$. This solution means there is at least one maximum and one minimum, hence one peak. We can find additional roots by solving for the other term in Equation (S9), which yields

$$t = \pm\frac{1}{\omega}\arccos\left[\frac{-a_1 a_2\, g_1\, g_2 \pm \sqrt{a_1{}^2 a_2\, g_1\, g_2 (a_1 + a_1 g_2 - a_2 g_2)}}{a_1{}^2 a_2\, g_1\, g_2}\right]. \tag{S10}$$

In the special case where the basal activities are set to zero and $A_1 = 1$, Equation (S10) simplifies to

$$t = \pm\frac{1}{\omega}\arccos\left[-1 \pm \frac{2}{\sqrt{\frac{A_2}{K_{M,1} \cdot K_{M,2}}}}\right]. \tag{S11}$$

Equation (S9) has only real positive roots when the argument of $\arccos$ is in the range [-1, 1], and so the condition $\frac{A_2}{K_{M,1} \cdot K_{M,2}} > 1$ must be verified. So long as the Michaelis-Menten constants are smaller than the maximal value of the input oscillators, which is a reasonable assumption, then that condition is verified. If, for example, $A_2 = A_1 = 1$ and $K_{M,1} = K_{M,2} = 1/2$, the whole set of solutions is $t = \left\{\frac{2\pi n - \frac{\pi}{2}}{\omega}, \frac{2\pi n}{\omega}, \frac{2\pi n + \frac{\pi}{2}}{\omega}, \frac{\pi + 2\pi n}{\omega}\right\}, n \in \mathbb{Z}$, and so the system generates two peaks of activity (and two troughs) in each circadian cycle. In this special case, the peaks are uniformly separated by 12 hours, but different parameterisations can tune the peak-to-peak distances to be non-uniform (such as we observed experimentally), and even to be so small as to effectively merge the double peak into a single peak.

This calculation only demonstrates that the production rate can easily exhibit double peaks. This is a necessary but not sufficient condition to produce double peaks of *expression*. If a double peak in production rate is too subtle, then the double peak will either be obscured or become a shoulder at the expression level, unless the dilution rates and other kinetic parameters are fast enough, or, alternatively, the Hill exponents are raised. These parameters can easily be tuned in the full model (Equations (S3-S5)).

2. Relation of oscillatory Hill equation dynamics to models with multiplication of sinusoidal terms.

A non-linear interaction between two or more periodic signals generally introduces second harmonics (Franken et al, 1961; Westermark & Herzel, 2013). In gene networks, typical sources of

non-linearities are cooperative binding and combinatorial regulation (Korenčič et al, 2012; Westermark & Herzel, 2013). These mechanisms can be phenomenologically modelled by Hill functions and products of input time variables. In the denominator of equation (S7) one finds the term $u_1.u_2$, which we can write as a product of two sinusoidal terms (S1), such that

$$u_1.u_2 = \frac{b_1+\frac{1}{2}(A_1-b_1)\left(1+cos\left(\frac{2\,\pi\,t}{24}\right)\right)}{K_{M,1}}\ \frac{b_2+\frac{1}{2}(A_2-b_2)\left(1+cos\left(\frac{2\,\pi\,t}{24}\right)\right)}{K_{M,2}}. \qquad \text{(S12)}$$

Using the trigonometric identity $2\big(cos(\omega\ t)\big)^2 = 1 + cos(2\ \omega\ t)$, (S12) becomes

$$u_1.u_2 = \text{Constant} + \frac{(A_1A_2-b_1b_2)}{2\,K_{M,1}\,K_{M,2}}cos\left(\frac{2\,\pi\,t}{24}\right) + \frac{(A_1-b_1)(A_2-b_2)}{8\,K_{M,1}\,K_{M,2}}cos\left(\frac{2\,\pi\,t}{12}\right), \qquad \text{(S13)}$$

where the last term is the second harmonic responsible for frequency doubling.

In the general case where the production rate is given by

$$f = V\frac{(u_1)^{h_1}}{1+(u_1)^{h_1}+(u_2)^{h_2}+(u_1)^{h_1}\,(u_2)^{h_2}}, \qquad \text{(S14)}$$

we can expand each of the input terms $(u)^h$, such that

$$(u)^h = \sum_{k=0}^{h}\binom{h}{k}\Gamma^{h-k}\ \mathrm{z}^k\left(cos\left(\frac{2\,\pi\,t}{24}\right)\right)^k, \qquad \text{(S15)}$$

where $\Gamma = \frac{(A+b)}{2\,K_M}$ and $b = \frac{(A-b)}{2\,K_M}$. The sinusoidal term can be expressed as a combination of harmonics according to the following general trigonometric identity:

$$\big(cos(\omega\ t)\big)^k = \begin{cases} \frac{2}{2^k}\sum_{q=0}^{\frac{k-1}{2}}\binom{k}{q}cos\big((k-2\,q)\omega\ t\big), & \text{if } k \text{ is odd} \\ \frac{1}{2^k}\binom{k}{\frac{k}{2}} + \frac{2}{2^k}\sum_{q=0}^{\frac{k}{2}-1}\binom{k}{q}cos\big((k-2\,q)\omega\ t\big), & \text{if } k \text{ is even} \end{cases}. \qquad \text{(S16)}$$

Discarding all terms beyond the second harmonic, (S15) becomes

$$(u)^h \approx \underbrace{\sum_{k\ \mathrm{odd}}^{h} \binom{h}{k} \binom{k}{\frac{k-1}{2}} \Gamma^{h-k}\, \mathrm{z}^k \frac{2}{2^k}}_{s} cos\left(\frac{2\,\pi\, t}{24}\right) + \underbrace{\sum_{k\ \mathrm{even}}^{h} \binom{h}{k} \binom{k}{\frac{k}{2}-1} \Gamma^{h-k}\, \mathrm{z}^k \frac{2}{2^k}}_{r} cos\left(\frac{2\,\pi\, t}{12}\right) +$$

$$+ \underbrace{\sum_{k\ \mathrm{even}}^{h} \binom{h}{k} \binom{k}{\frac{k}{2}} \Gamma^{h-k}\, \mathrm{z}^k \frac{1}{2^k}}_{\varepsilon}, \qquad \text{(S17)}$$

where s represents the amplitudes of all 24 hour components, r represents the amplitudes of all 12 hour components, i.e., the second harmonic, and ε is a constant. Replacing into (S14), we can derive the general form of the production rate in terms of the first and second harmonics:

$$f \approx V \frac{\varepsilon_1 + s_1 cos\left(\frac{2\,\pi\, t}{24}\right) + r_1 cos\left(\frac{2\,\pi\, t}{12}\right)}{1+\varepsilon_1+\varepsilon_2+\varepsilon_1\varepsilon_2+\frac{s_1 s_2}{2}+\frac{r_1 r_2}{2}+\left(s_1+s_2+\varepsilon_1 s_2+\varepsilon_2 s_1+\frac{s_1 r_2}{2}+\frac{s_2 r_1}{2}\right)cos\left(\frac{2\,\pi\, t}{24}\right)+\left(r_1+r_2+\varepsilon_1 r_2+\varepsilon_2 r_1+\frac{s_1 s_2}{2}\right)cos\left(\frac{2\,\pi\, t}{12}\right)}.$$

(S18)

References:

Chabot JR, Pedraza JM, Luitel P, van Oudenaarden A (2007) Stochastic gene expression out-of-steady-state in the cyanobacterial circadian clock. *Nature* **450:** 1249-1252

Franken P, Hill AE, Peters C, Weinreich G (1961) Generation of optical harmonics. *Phys Rev Lett* **7:** 118-119

Korenčič A, Bordyugov G, Rozman D, Goličnik M, Herzel H (2012) The interplay of cis-regulatory elements rules circadian rhythms in mouse liver. *PLoS One* **7:** e46835

Westermark PO, Herzel H (2013) Mechanism for 12 hr rhythm generation by the circadian clock. *Cell Rep* **3:** 1228-1238
