# Supplementary material for: Frequency doubling in the cyanobacterial circadian clock
Source: Mol Syst Biol. 2016 Dec 22;12(12):896. doi: 10.15252/msb.20167087 (PMC5199125; doi:10.15252/msb.20167087)
Supplement: Supplementary file 2 — Expanded View Figures PDF [file MSB-12-896-s002.pdf]

Expanded View Figures

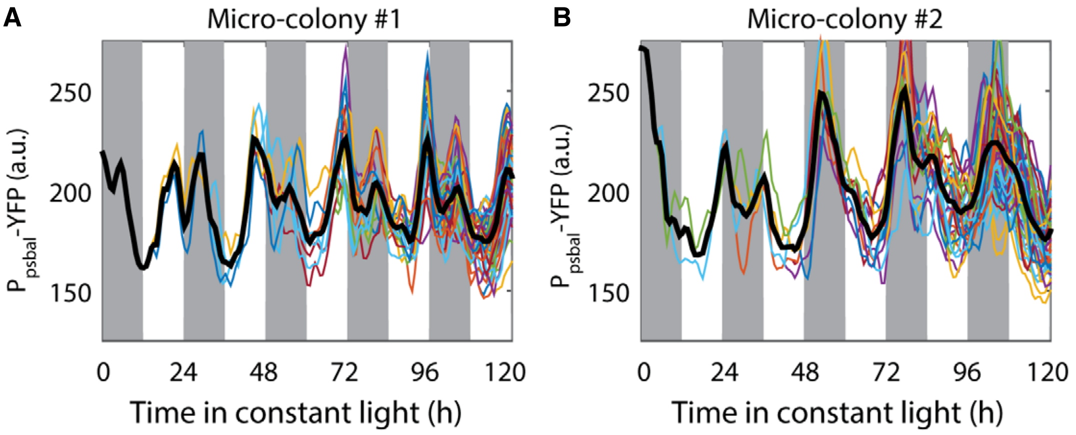

Figure EV1. Single-cell lineages from two movies of *psbAI* expression show two-peak oscillations.

A Time traces of P_{psbAI}-YFP reporter strains grown under low light (ca. 15 μE m^{-2} s^{-1} cool white light). Individual lineages show the existence of a secondary peak following the main (dusk timed) peak of expression. Cells remain synchronised, allowing double peak to be observed in the mean trace (black line).

B In a different movie, due to desynchronisation, the double peak is less apparent in the mean trace (black line).

Data information: In (A) and (B), each line under the black mean trace represents one single-cell lineage.

© 2016 The Authors

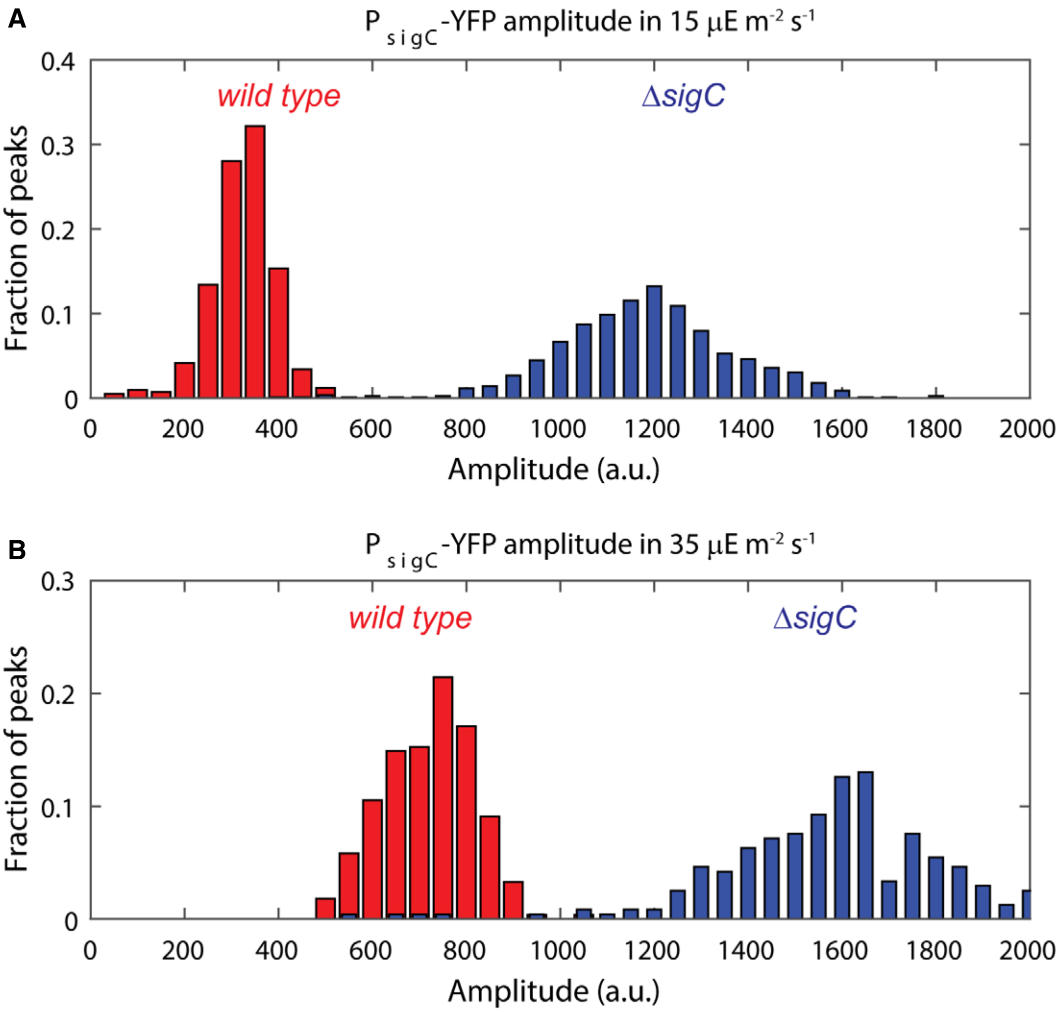

Figure EV2. P_{sigC}-YFP levels are upregulated in high light, suggesting a reduced functionality of SigC in higher light conditions.

A Histogram of P_{sigC}-YFP expression in wild-type (red) and *sigC* deletion mutant (blue) under ca. 15 μE m^{-2} s^{-1} cool white light. For the wild type, 664 cells from nine movies were collected, whereas for the *sigC* deletion strain, 1,084 cells from six movies were collected.

B Histogram of P_{sigC}-YFP expression in wild-type (red) and *sigC* deletion mutant (blue) under ca. 35 μE m^{-2} s^{-1} cool white light. P_{sigC}-YFP in wild type is upregulated by twofold between light conditions. A smaller fold change of 1.2 is seen in the *sigC* deletion mutant. In the higher light condition, 1,003 cells from two movies were collected for the wild-type strain, whereas 920 cells from two movies were collected for the *sigC* deletion strain.

Source data are available online for this figure.

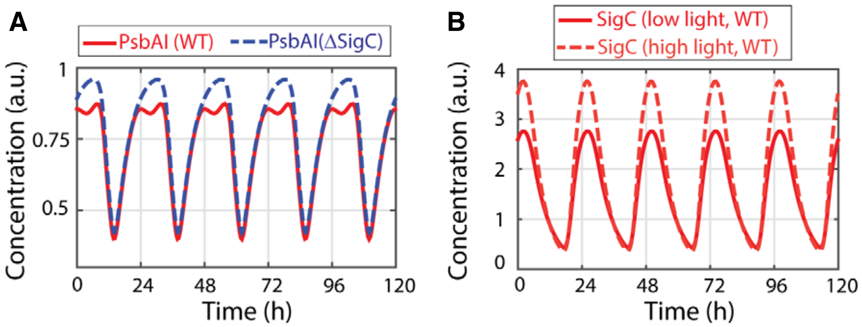

Figure EV3. Simulations of reduced SigC activity at high light qualitatively match experimental data.

A Increasing the deactivation rate of SigC by two orders of magnitude produces only a minor double peak in PsbAI expression. This is comparable to that observed experimentally in higher light conditions (Fig 5A).

B The reduction in SigC activity in simulations results in an increase in SigC expression, which qualitatively matches experimental results (Fig EV2). The traces represent the sum of the concentration of the two forms of SigC we considered in the model (see Section II in Appendix).

 © 2016 The Authors

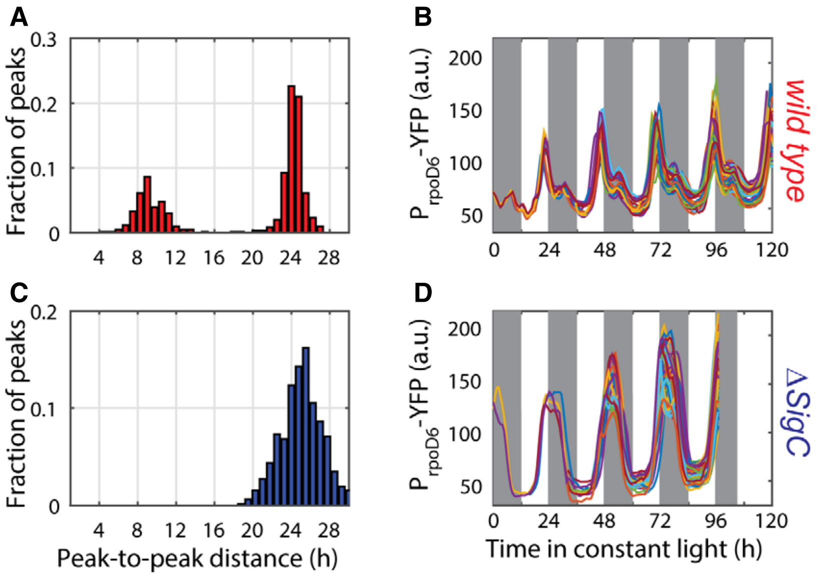

Figure EV4. Double peaks in P_{rpoD6}-YFP expression are dependent on *sigC*.

A Measure of the distance between the first peak in each circadian cycle and the following peak shows only a subfraction of cells display a double peak. 947 cells from 12 movies (with up to 272 cells per time point) were collected.

B Single-cell lineages of a representative movie show that all lineages show either a second peak, or a shoulderlike feature, in P_{rpoD6}-YFP levels.

C In the *sigC* deletion mutant, the two-peak oscillations are abolished. 1,352 cells from seven movies (with up to 502 cells per time point) were collected.

D Single-cell lineages of a representative movie show a single peak of P_{rpoD6}-YFP.

Source data are available online for this figure.

© 2016 The Authors
