# Supplementary material for: Frequency doubling in the cyanobacterial circadian clock
Source: Mol Syst Biol. 2016 Dec 22;12(12):896. doi: 10.15252/msb.20167087 (PMC5199125; doi:10.15252/msb.20167087)
Supplement: Supplementary file 3 — Movie EV1 [file MSB-12-896-s003.zip › MovieEV1/MovieEV1Legend.txt.rtf]

Movie EV1: Time‐apse movie of strain 7942_S19 carrying the reporter PpsbAI‐FP in a wild type background. A two peak oscillation in psbAI expression can be seen at the level of single cells. The time between frames in this movie is 45 minutes.
