# Supplementary material for: Frequency doubling in the cyanobacterial circadian clock
Source: Mol Syst Biol. 2016 Dec 22;12(12):896. doi: 10.15252/msb.20167087 (PMC5199125; doi:10.15252/msb.20167087)
Supplement: Supplementary file 4 — Movie EV2 [file MSB-12-896-s004.zip › MovieEV2/MovieEV2Legend.rtf]

Movie EV2: Time‐apse movie of strain 7942_A4 carrying the reporter PsigC‐FP in a wild type background. These cells exhibit single peak oscillations in sigC expression. The time between frames in this movie is 45 minutes.
